# Ultrasound-Proven Severe Synovitis Induced by PD-1 Inhibitor Therapy in a Patient Predisposed to Seronegative Inflammatory Arthritis

**DOI:** 10.1155/2019/7340692

**Published:** 2019-07-24

**Authors:** Shin-ya Kawashiri, Naoki Iwamoto, Kojiro Ohba, Atsushi Kawakami

**Affiliations:** ^1^Departments of Community Medicine, Nagasaki University Graduate School of Biomedical Sciences, Nagasaki, Japan; ^2^Departments of Immunology and Rheumatology, Nagasaki University Graduate School of Biomedical Sciences, Nagasaki, Japan; ^3^Departments of Urology, Nagasaki University Graduate School of Biomedical Sciences, Nagasaki, Japan

## Abstract

A 71-year-old Japanese woman was treated with programmed cell death protein-1 (PD-1) inhibitor, nivolumab, for renal cell carcinoma with lung metastasis. Although she had been treated with antirheumatic drugs from 40 to 60 years old by the diagnosis of seronegative inflammatory arthritis, treatment was discontinued based on her achievement of remission. She developed severe polyarthralgia after the administration of nivolumab. Severe synovitis with remarkable power Doppler signals was detected by ultrasound in multiple joints and tendons, and her serum levels of proinflammatory cytokine were remarkably elevated. Nevertheless, her arthritis disappeared after the discontinuation of nivolumab and treatment with a glucocorticoid without antirheumatic drugs. The use of PD-1 inhibitor may be restricted in patients predisposed to arthritis. Alternatively, a close monitoring of these patients by rheumatologists is necessary to identify predictable flares.

## 1. Introduction

In recent years, immune checkpoint inhibitors (ICIs) targeting programmed cell death protein-1 (PD-1) and cytotoxic T-lymphocyte-associated protein 4 (CTLA-4) have been introduced in patients with various cancers. The number of reports about immune-related adverse events (IrAEs) including inflammatory arthritis induced by ICIs has been increasing [1–6]. The incidence of inflammatory arthritis as an IrAE was estimated to be 1.3%–3.8% [2, 3]. We present a patient predisposed to seronegative inflammatory arthritis and induced ultrasound-confirmed severe synovitis by PD-1 inhibitor.

## 2. Case Presentation

A 71-year-old Japanese woman had a right nephrectomy for renal cell carcinoma 20 months prior to the present examination. Lung metastasis was observed 5 months prior to the present examination. She had undergone treatment with sunitinib (a multitargeted receptor tyrosine kinase inhibitor), but this treatment was discontinued due to severe liver dysfunction and pancreatitis. She was treated with nivolumab (PD-1 inhibitor) 2 months before the present examination. Because she developed severe polyarthralgia of bilateral fingers and wrists and then of her neck and bilateral shoulders after the administration of nivolumab, she was referred by our hospital's Department of Urology to our Department of Immunology and Rheumatology. She had been diagnosed as having seronegative inflammatory arthritis at the age of 40 years and was treated for RA for 20 years; treatment with antirheumatic drugs (unknown) was discontinued based on her achievement of remission.

On examination, she had eight swollen and six tender joints including the metacarpophalangeal (MCP), proximal interphalangeal (PIP), wrist, and shoulder joints. Laboratory findings showed increased C-reactive protein (CRP, 109 mg/l, normal range 0–30 mg/l), erythrocyte sedimentation rate (ESR, 104 mm/hr, normal range <20 mm/hr), and matrix metalloproteinase-3 (969 ng/ml, normal range 17.3–59.7 ng/ml). Both IgM-rheumatoid factor (RF) and anticyclic citrullinated peptide antibodies (ACPA) were negative. The titer of antinuclear antibodies (ANA) was ×160 (cytoplasmic pattern). Some serum biomarkers measured by the Bioplex Pro Human Cytokine assay (BioRad, Hercules, CA) were increased: IL-1*β*, 22.7 pg/ml (normal range, 0.3–1.5 pg/ml); IL-6, 21.3 pg/ml (normal range, 0.1–1.9 pg/ml); and TNF*α*, 20.6 pg/ml (normal range, 5.3–8.9 pg/ml) [7].

**Figure 1:**
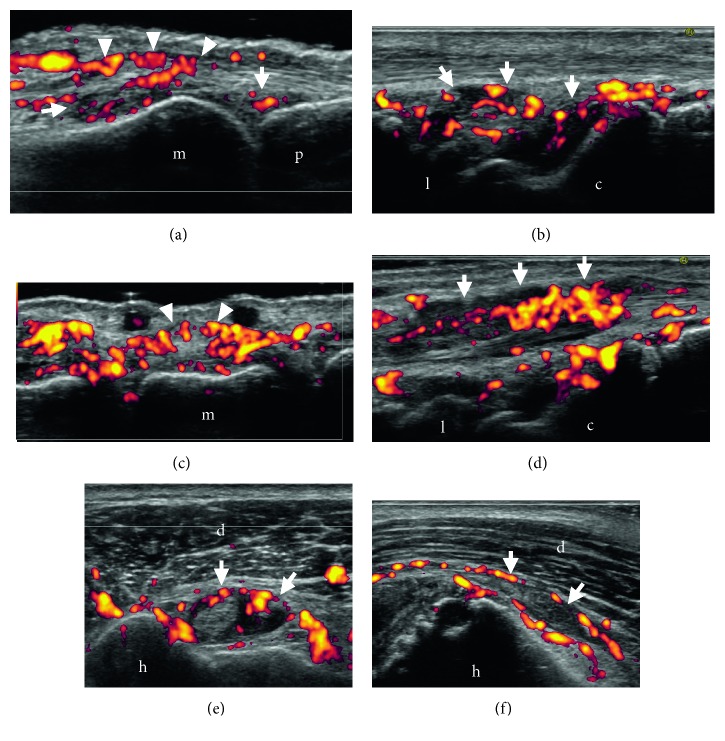
Severe synovitis detected by ultrasonography at 3 months after the introduction of nivolumab. (a) Articular synovitis (arrow) and peritendinitis of digital extensor tendon (arrowhead) in the 2nd metacarpophalangeal (MCP) joint (dorsal aspect, longitudinal view). (b) Articular synovitis (arrow) in a radiocarpal-intercarpal joint (dorsal aspect, longitudinal view). (c) Peritendinitis of digital extensor tendon (arrowhead) in MCP joint (dorsal aspect, transverse view). (d) Tenosynovitis (arrow) of the fourth compartment (extensor indicis and extensor digitorum communis) of the extensor tendons (dorsal aspect, longitudinal view). (e) Tenosynovitis (arrow) of the long head of the biceps (transverse view). (f) Subdeltoid bursitis (arrow) (longitudinal view). (c) Capitatum. (d) Deltoid muscle. (h) Humerus. (l) Lunatum. (m) Media. (p) Proximalis.

Plain X-rays of bilateral hands and shoulders showed no joint space narrowing and no bone erosion. Ultrasound detected severe articular synovitis represented by synovial hypertrophy with remarkable power Doppler (PD) signals in bilateral MCP (Figure 1(a)) and wrists (Figure 1(b)), PD-positive peritendinitis of digital extensor tendons (Figure 1(a) and 1(c)), tenosynovitis of carpal extensor tendons (Figure 1(d)) and digital flexor tendons, tenosynovitis of the long head of the biceps (Figure 1(e)), and subdeltoid-subacromial bursitis (Figure 1(f)). Erosion was not detected by ultrasound.

We concluded that her symptoms were due to inflammatory arthritis induced by nivolumab. Treatment was initiated with oral prednisolone 10 mg daily without antirheumatic drugs. The nivolumab treatment was discontinued because it had no effect on the patient's tumor; her symptoms improved immediately and have not relapsed regardless of the discontinuation of prednisolone.

## 3. Discussion

PD-1 inhibitor therapy has induced ultrasound-confirmed severe synovitis in a patient predisposed to seronegative inflammatory arthritis. A recent case report described RA recurrence in patients with preexisting RA in remission after they received nivolumab [6]. PD-positive synovitis was confirmed by ultrasound in some cases with inflammatory arthritis induced by ICIs [2, 3]. In our patient's case, severe synovitis with remarkable PD signals was detected by ultrasound in multiple joints and tendons and her serum levels of proinflammatory cytokine were remarkably elevated. Nevertheless, her arthritis disappeared after the discontinuation of nivolumab and treatment with a glucocorticoid without antirheumatic drugs.

ICIs act by blocking negative costimulatory molecules on T cells, antigen-presenting cells, and tumor cells. The blockade of these inhibitory molecules allows for unchecked T-cell activation and a subsequent immune response targeting tumors [4]. Recent reports showed that PD-1 suppresses autoimmune arthritis by inhibiting the Th17 response [8] and that PD-1 blockade augments the Th17 response in collagen-induced arthritis (CIA) mice [9]. Our patient might have reactive properties of the Th17 response.

In patients treated with PD-1 inhibitor, preexisting autoimmune or inflammatory disease was reported to be associated with a significantly increased risk of IrAEs and flares [5]. Autoreactive T cells may be susceptible to activation by ICIs in autoimmune or inflammatory diseases, arising from a breakdown in immunological self-tolerance. Therefore, the use of ICIs may be restricted in patients predisposed to inflammatory arthritis. Alternatively, a close monitoring of these patients by rheumatologists is necessary to identify predictable flares.

In conclusion, PD-1 inhibitor therapy has induced ultrasound-confirmed severe synovitis in a patient predisposed to seronegative inflammatory arthritis. Therefore, we may be careful to use PD-1 inhibitor in patients predisposed to inflammatory arthritis.

## Abbreviations

ICIs: Immune checkpoint inhibitors
IrAEs: Immune-related adverse events
MCP: Metacarpophalangeal
PD-1: Programmed cell death protein 1
PD: Power Doppler
PIP: Proximal interphalangeal.
